# Are exon 19 deletions and L858R EGFR mutations in non-small-cell lung cancer clinically different?

**DOI:** 10.1038/sj.bjc.6603564

**Published:** 2007-01-16

**Authors:** D B Costa, S Kobayashi

**Affiliations:** 1 Division of Hematology/Oncology, Beth Israel Deaconess Medical Center, Harvard Medical School, Boston, MA, USA

**Sir**,

Asahina *et al* (2006) report the second prospective trial of gefitinib as front-line therapy for epidermal growth factor receptor (EGFR)-mutant metastatic non-small-cell lung cancer (NSCLC). The impressive results of these initial 32 patients from two separate Japanese groups is unheard in NSCLC and shows the promise of patient selection for use of targeted therapy (Asahina *et al*, 2006; Inoue *et al*, 2006). However, it is still elusive if different types of EGFR tyrosine kinase mutations have different clinical impact. The two most common activating mutations seen in patients are exon 19 deletions and the exon 21 L858R. In the largest retrospective cohorts of patients from two United States centres who followed EGFR-mutant NSCLC patients given tyrosine kinase inhibitors (gefitinib or erlotinib) as first to third-line therapy, it was observed that patients with exon 19 deletions had a significantly improved time to progression and overall survival when compared with L858R patients (Jackman *et al*, 2006; Riely *et al*, 2006). In both reports, patients with exon 19 in-frame deletions had at least double the progression-free and overall survival of the group with the L858R mutation.

In the two Japanese prospective trials, the response rates for both mutations were not significantly different. Inoue *et al* (2006) reported response rates of 67 and 86% for exon 19 deletions and L858R mutants, respectively. In the current Asahina *et al* (2006) article in the *British Journal of Cancer*, the response rates were 83% for exon 19 deletions and 67% for L858R patients. The time to progression in the later trial seems to be similar in both different mutant cohorts and, of note, one of the patients with the most prolonged response (over 13 months) had an L858R mutation (Asahina *et al*, 2006).

Further data collection from the ongoing prospective trials of EGFR tyrosine kinase inhibitors (Asahina *et al*, 2006; Inoue *et al*, 2006; Paz-Ares *et al*, 2006) will determine if there is a real clinical difference in response and survival between the two most common EGFR mutations. The verdict is still out if EGFR activating mutations in NSCLC are not created equal.
